# Changes in the Expression of Some Genes With Metabolic, VLDL and Antioxidative Effects After the Addition of Essential Oil Mixture to Drinking Water in the Liver of Domestic Geese (*Anser anser Domesticus*)

**DOI:** 10.1002/vms3.70285

**Published:** 2025-03-03

**Authors:** Özlem Durna, Mustafa Hitit, Zafer Usta, Gültekin Yildiz

**Affiliations:** ^1^ Department of Animal Nutrition and Nutritional Diseases, Faculty of Veterinary Medicine Dicle University Diyarbakır Türkiye; ^2^ Department of Genetics Faculty of Veterinary Medicine Kastamonu University Kastamonu Türkiye; ^3^ College of Agriculture, Food and Natural Resources, Cooperative Agricultural Research Center Prairie View A&M University Prairie View Texas USA; ^4^ Department of Genetics, Faculty of Veterinary Medicine Burdur Mehmet Akif Ersoy University Burdur Türkiye; ^5^ Department of Animal Nutrition, and Nutritional Diseases, Faculty of Veterinary Medicine Ankara University Ankara Türkiye

**Keywords:** antioxidative, essential oil, geese, gene expression, metabolic, VLDL

## Abstract

Studies have shown that essential oils not only increase cell viability but also affect lipid metabolism in mammals. However, the extent to which these effects are realized in goose liver has not yet been fully elucidated. The object of research is to investigate the effects of four essential oil mixtures (juniper oil, mint oil, thyme oil, rosemary oil) on lipid metabolic gene expressions in goose. We measured mRNA levels of metabolic genes (*ACSBG2, ELOVL1, ELOVL2, CYP2Cl9, CYP2K1*), antioxidative gene (*SOD1*) and very low‐density lipoprotein triglyceride (VLDL) synthesis genes (*APOB, FOXO1, MTTP*), in goose (*Anser anser*) liver. Search groups were formed as C (control; no additives), EK1 (0.4 mL/L essential oil mixture supplemented) and EK2 (0.8 mL/L essential oil mixture supplemented). The relative expression levels of genes in the liver were measured using RT‐qPCR. β‐Actin was used as reference gene control for normalization of qPCR data. As a result, essential oil supplementation downregulated metabolic genes compared to the control group. *APOB* gene among VLDL genes was significantly downregulated. Antioxidative effect gene was downregulated in parallel with the others. This indicates that essential oil intake with drinking water downregulates the genes involved in lipid metabolism in goose liver. Our data show that essential oils have a significant effect on the regulation of genes and pathways involved in hepatic lipid metabolism.

## Graphical abstract

Our data show that essential oils have a significant effect on the regulation of genes and pathways involved in hepatic lipid metabolism. According to the results obtained in our study, increasing the intake of essential oils with food decreased *APOB* gene expression in the liver, and it is thought that essential oils intake in cholesterol‐related diseases in humans will reduce cholesterol and cardiovascular diseases.

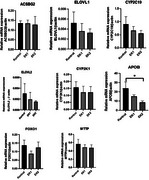

## 1 Introduction

The use of antibiotics in animal nutrition is prohibited because of the risk of resistant bacteria that may pose a risk to human health (Orinetha et al. 2022). Accordingly, studies on the development of alternative feed additives have gained momentum. In this regard, herbal extracts are recognized as one of the effective solutions (Bilal et al. 2008). In the light of this knowledge, essential oil supplementation in feed or water is a new strategy to improve animal health. It has been reported that essential oil composites have antiparasitic, antimicrobial, antiviral, antimycotic, antioxidant and anti‐inflammatory effects; stimulate the digestive system of animals and increase the efficiency of digestive enzymes (Bilal et al. 2008, Durna Aydin et al. 2022).

There are some studies on the effects of essential oil and fat metabolisms components on poultry (Xing and Li 2023). In studies conducted in quails and broilers, it was determined that essential oils improved live weight gain and feed utilization compared to the control group (Alcicek et al. 2004; Denli et al. 2004). In studies carried out in laying hens, it was observed to increase egg yield (Bilal et al. 2008; Bölükbaşi and Erhan 2007).

Juniper (*Juniperus communis* L.) is an aromatic bush with a major therapeutic effect in the treatment of diseases (Raina et al. 2019). The plant is rich in aromatic oils, invert sugars, resins, organic acid, alkaloids, tannins, gums, lignins and wax (Raina et al. 2019). Juniper essential oil has antioxidant, antibacterial, antiviral and antifungal activities (Raina et al. 2019). Studies have also detected anti‐inflammatory, cytotoxic, hypoglycemic and hypolipidemic effects of juniper berries (Raina et al. 2019).

*Lamiaceae*, also known as the mint family, is an extensive family of flowering plants (Salehi et al. 2019). The plants of this family are usually shrubs or herbs that contain aromatic mixtures such as essential oils in their leaves or flowers (Salehi et al. 2019). Mint oil mixtures are generally used as antispasmodic, astringent, anti‐proliferative, anti‐inflammatory, antiviral and regulator of oil secretions (Salehi et al. 2019).

*Thymus vulgaris* (thyme) is an aromatic plant from the Lamiaceae family and has effects as both a pharmaceutical and therapeutic agent (Gumus et al. 2017). It is reported that when added to poultry feed, due to the phenolic compounds it contains, thyme increases feed intake as well as the secretion of endogenous digestive enzymes and strengthens the immune system (Gumus et al. 2017).

Rosemary (*Rosmarinus officinalis*, L.) is an aromatic plant from the Lamiaceae family, originating from the Mediterranean region (Nieto et al. 2018). Rosemary extracts are also used in food conservation because they prevent oxidation and microbial contamination. Rosemary extracts as preservatives offer various technological advantages and benefits to customers (Nieto et al. 2018).

The liver, the second largest organ of the body, has a wide range of metabolic activities from the processing of nutrients taken with solid and liquid foods to the removal of harmful substances from the body. To determine the metabolic effects of essential oils in the liver, we studied nine gene pathways with different functions.

Apolipoprotein B (*APOB*) is produced by the liver and secreted by the liver in the form of very low‐density lipoproteins (VLDL) (Sidiropoulos et al. 2007). *APOB* is the key protein of low‐density lipoproteins (LDL) and also roles as a ligand for the LDL receptor, mediating the approval and purification of LDL from plasma (Sidiropoulos et al. 2007). Forkhead box O1 (*FoxO1)* is a transcription factor that plays an important part in the hepatic VLDL gathering and secretion (D.D. Liu, Han et al. 2016). *FoxO1* stimulated microsomal trigliyceride transfer protein (MTTP) expression, increased APOB secretion and increased VLDL production by binding and stimulating MTTP promoter activity. (D.D. Liu, Han et al. 2016).

The cytochrome P450 (*CYP*) gene family encodes enzymes involved in the metabolism of certain foreign chemicals such as drugs; arachidonic acid metabolism; cholesterol, sterol and bile acid biosynthesis; steroid and vitamin D synthesis and catabolism (Nelson et al. 2004). The *CYP* superfamily is therefore a crucial gene for survival (Almeida et al. 2016). Elongation of very long‐chain fatty acids (*ELOVL*) proteins play an important role in determining the length of a fatty acid (Ferraz et al. 2022). The *ELOVL1* exhibits a substrate favourite for saturated and monounsaturated fatty acids, while *ELOVL2* usages polyunsaturated fatty acids as substrates (Ferraz et al. 2022). Fatty acid activation is catalyzed by the frequency‐limiting effect of Acyl–CoA synthases (Lopes‐Marques et al. 2018). Accordingly, severeal families of Acyl‐CoA enzymes have been previously identified and classified according to the length of the fatty acid chain they process (Lopes‐Marques et al. 2018). ACSBG enzymes, also known as lipidosin, activate fatty acids between C16 and C24. Currently, two members of the *ACSBG* gene family have been identified in mammals, *ACSBG1* and *ACSBG2* (Lopes‐Marques et al. 2018).

Superoxide dismutase 1 *(SOD1*), a copper‐ and zinc‐containing *SOD* (CuZn‐SOD), restricts mostly to oxytoplasmic and nuclear sections (Folz et al. 1997).

The aim of this study was to gain insight into the effect of some essential oils (juniper oil, peppermint oil, thyme oil and rosemary oil) on the regulation of some of the abovementioned genes involved in hepatic metabolic processes in geese and to determine the expression of genes that may have metabolic, HDL, LDL, VLDL and antioxidative roles in geese drinking water supplemented with the abovementioned oils.

## 2 Material and Methods

### 2.1 Animals, Experimental Design and Feed

Ethical approval for study was received from the Animal Experiments Local Ethics Committee of Kafkas University (No: 2019–127). The animals were fed with a basal diet based on Table 1. All diets were specified according to NRC standards (NRC 1994). Nutrient analyses of the feed were applied according to AOAC (2000). A total of 108 chicks (3 days old) were randomly divided into three groups (36 chicks in each group), and six chicks randomly selected from these three groups formed the control, EK1 and EK2 groups. In total, 18 chicks were used for the genetic study. The research groups were designed as follows: C (control; no additives), EK1 (0.4ml/L essential oil blend supplement in drinking water) and EK2 (0.8ml/l essential oil blend supplement in drinking water). The duration of the experiment was 13 weeks. Durind the first 4 weeks of this period, the animals were given chick period feed. During the next 9 weeks, the geese were fed in the pasture conditions of Kars province. The essential oil mixture (Mintofarm) used in the study was obtained from a private firm. The preparation of Mintofarm used in the study is shown in Table 2.

**TABLE 1:** Composition of diets in experiment (%)^a^.

Feed materials	Percentage
Corn	56.35
Soybean meal (CP,46%)	36.10
Corn gluten (CP, 60%)	4.35
Limestone	1.45
Dicalcium phosphate	1.00
DL‐methionine	0.08
l‐Lysine hydrochloride	0.07
Vitamin‐mineral premix^b^	0.40
Salt	0.20
Total	100.00
The calculated value	
Crude protein (%)	23.00
ME (kcal/kg)	2909.33
Ca (%)	0.90
Total P (%)	0.59
Analysis Values	
ME (kcal/kg)	2915.25
Crude protein (%)	23.11
Ca (%)	1.01
Total P (%)	0.49

^a^ As‐fed basis.

^b^ Vitamin‐mineral premix provided per kg diet: Vit. A 8000 IU, Vit. D3 1000 IU, Vit. E 20 IU, Vit. K 0.5 mg, Vit. B1 3 mg, Vit. B2 9 mg, Vit. B6 7 mg, Vit. B12 0.03 mg, niacin 35 mg, d‐pantothenic acid 10 mg, folic acid 0.55 mg, biotin 0.18 mg, Fe 100 mg, Cu 8 mg, Zn 100 mg, Mn 120 mg, I 0.7 mg, and Se 0.3 mg.

**TABLE 2:** Formulation essential oil mixture used in the study (%).^a^

Product combination	Percentage
Juniper oil	2
Mint oil	2
Thyme oil	2
Rosemary oil	2
Surfactants and stabilizers	15
Water (transporter)	77

^a^ Mintofarm.

### 2.2 RNA Isolation

Total RNA isolation and quality control from liver tissue samples were performed using the protocol from Kurar et al. 2010. A total of 30 mg of liver sample was cut with a scalpel and homogenized in 1 mL of TRIzol (Thermo Fisher Scientific, TRIzol Reagent, USA) with the help of a mechanical homogenizer. Note that 200 µL of chloroform was added to the 1000 µL suspension taken into the Eppendorf tube and mixed by vortex. The samples, which were kept at room temperature for 10 min, were centrifuged for 15 min at a speed of 13,000 *g* in a cooled centrifuge at +4°C. The resulting supernatant was separated into another Eppendorf tube without disturbing the phases. Note that 500 µL isopropanol (Sigma‐Aldrich) was added to 350–400 µL supernatant, the mixture was mixed by turning it upside down, and it was kept at room temperature for 10 min; then, the RNA pellet formed was precipitated at the bottom by centrifugation at 13,000 *g* speed for 10 min in a cooled centrifuge. After removing the supernatant part, washing with cold ethanol three times (70%, 70% and 95%, respectively) was done at +4°C at 7500 *g* speed for 5 min. After the RNA pellet was dried, it was poured into 50 µL diethyl pyrocarbonate‐treated water (DEPC‐dH_2_O) has been resolved. The quantity and quality of the RNA samples were determined using the Colibri Microvolume spectrophotometer. In order to determine the quality of the RNA samples, the quality of the RNA bands stained with ethidium bromide after 1% agarose gel electrophoresis by placing 5 µL of 6X loading dye on RNA at a concentration of 1 µg/10 µL was checked visually on a UV‐transluminator and in the imaging system. Total RNA samples were stored at −80°C. In order to eliminate possible gDNA contamination, DNAse‐I enzyme reaction was applied according to the manufacturer's protocol. According to this protocol, 1 µL of DNAse‐I reaction mixture and 1 µL of DNAse‐I enzyme were placed on 1 µg/20 µL of total RNA and kept at 37°C for 30 min. In order to stop the enzymatic reaction, it was incubated for 10 min at 65°C with the addition of 1 µL of ethylenediaminetetraacetic acid.

### 2.3 cDNA Synthesis

After genomic DNAse‐I digestion for Real‐time quantitative PCR (RT‐qPCR) analysis, 1–2 µg of RNA for cDNA processing from RNA, 1 µL of oligo dT and 1 µL of random hexamer primer mix were kept at 65°C for 5 min for polymerization. Then, mix (I‐script, BioRad, USA) was created for reverse transcription, and 8 µL of reverse transcriptase (RT) enzyme mix was added to the mixture after incubation. In order to complete the reverse transcription process, it was incubated at 42°C for 60 min, and the process was terminated by keeping it at 70°C for 10 minutes.

### 2.4 Gene Expression by Quantitative Polymerase Chain Reaction

In the study, using sequence information defined over metabolic genes (*ACSBG2, ELOVL1, ELOVL2, CYP2Cl9, CYP2KI*), VLDL genes (*APOB, FOXO1, MTTP*), antioxidative gene (*SOD1*) and housekeeping gene (*β‐actin*) were designed according to the assembly of *Anser cyngoides* with the accession of GCF_002166845.1 by using Primer3 from the sequences in NCBI gene database or from published primer sequence. The sequences of the metabolic gene primers used in our study, as forward and reverse primers, are given in Table 3.

**TABLE 3:** Primer sequences for real‐time PCR in metabolic genes.

Gene name	Forward primer (5'‐3')	Reverse primer	Reference
FoxO1	CATCCCTTCAGTCTGGTCAA	GAAAGGCTGGGTAAAGTAG	Han et al. (2015)
MTTP	CCCGATGAAGGAGAGGAA	AAAATGTAACTGGCCTGAGT	Han et al. (2015)
APOB	CTCAAGCCAACGAAGAAG	AAGCAAGTCAAGGCAAAA	Han et al. (2015)
ACSBG2	GAAGGGAATTTCAGCAGTC	GGGAAAGTTTCTACCACGT	Tang et al. (2018)
CYP2C19	CTCAGCAGGGAAGCGAATA	GAGGTTTCGTGCCCATCAG	Tang et al. (2018)
CYP2K1	GCTCTTACACTCGGGAACT	TCTCAACCACAGGGCATAG	Tang et al. (2018)
ELOVL1	ACCAACGGCAAGGTCAAAG	CCAGGGACAAGTCGGTTCA	Tang et al. (2018)
ELOVL2	CTGCACTAGGTGGGCGTTAT	ACTCCAAAGGTGGTTTCGTT	Tang et al. (2018)
SOD1	GTCATCCACTTCCAGCAGC	CCCTTTACCCAGGTCATCG	Tang et al. (2018)
β‐Actin^a^	CAACGAGCGGTTCAGGTGT	TGGAGTTGAAGGTGGTCTCG	Han et al. (2015)
18S^a^	TTGGTGGAGCGATTTGTC	ATCTCGGGTGGCTGAACG	Han et al. (2015)

^a^ Reference gene.

According to the kit protocol, 10 µL of Sso Advanced SYBR Green PCR Master Mix (Biorad, USA), 5 pMol of primer and ddH2O were added to 4 µL cDNA converted from 1 to 2 µg RNA, and the total volume was completed to 20 µL. RT‐qPCR was performed with Rotor‐Gene Q (QIAGEN). RT‐qPCR analysis was performed for 40 cycles at 94°C for 15 s, at 60°C for 30 s, and at 70°C for 30 s after incubation at 95°C for 15 min. Melting curve analysis to demonstrate specific binding of the primer: 1 min at 95°C, then increased by 0.8°C per second from 60 to 95°C. The expression profiles of the obtained cDNAs were monitored using RT‐qPCR (Atli et al. 2010). Data from RT‐qPCR were recorded as Cq. The same amount of mix without template (ddH_2_O) was used as negative control. The products obtained from RT‐qPCR were confirmed to be the correct product by melting curve analysis and by observing the band sizes by running on agarose gel. The technique was repeated at all stages in RT‐qPCR.

### 2.5 RT‐q(PCR) Data Analysis

The primary efficacy was calculated using five data points from the kinetic curve graph of the exponential phase of the graph of fluorescence values log transformed according to the method defined by Schefe et al. (2006). If the efficiency was found to be 100%, mRNA RT‐qPCR data were normalized with the method of Livak and Schmittgen (2001). If the efficiency was not 100%, normalization was performed using the determined efficiency coefficient. RNA RT‐qPCR relative gene expression was confirmed by assay design (F‐test) analysis methods. mRNA RT‐qPCR gene expression data were statistically analyzed with the SPSS (SPSS15.0 for Windows; Armonk, NY, USA) software using one‐way factorial analysis of variance (ANOVA) method Tukey multiple comparison test (SPSS, 2015).

## 3 Results

### 3.1 Metabolic Gene Expression

**FIGURE 1:**
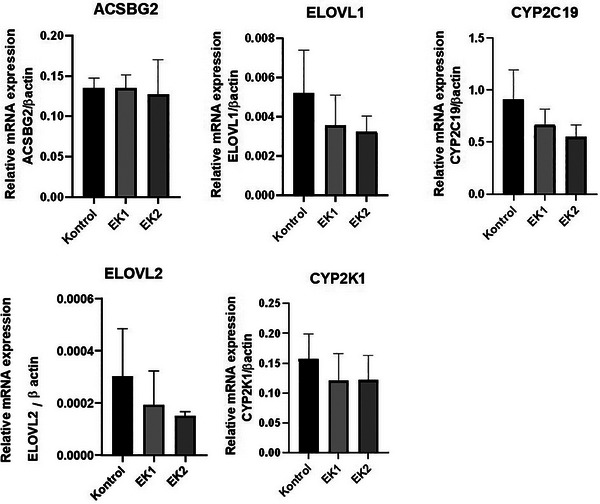
Expression levels of metabolic genes mRNA in the control, EK1 and EK2 groups.

In our study, involved metabolic mRNA gene expressions were investigated in the control, EK1 and EK2 groups. The expression levels of metabolic genes decreased in *ELOVL1, ELOVL2, CYP2C19* and *CYP2K1* genes in the EK1 group compared to the control group (Figure 1). In the EK2 group, all of metabolic genes were downregulated compared to the control group (Figure 1). *ACSBG2* gene was increased in the EK1 group compared to the control group (Figure 1). *CYP2K1* gene expression decreased in the EK1 and EK2 groups compared to the control group and was stably expressed between EK1 and EK2. On the other hand, the increases and decreases in the regulation in the metabolic genes group are not statistically significant (*p* > 0.05) (Figure 1).

### 3.2 VLDL Gene Expression

**FIGURE 2:**
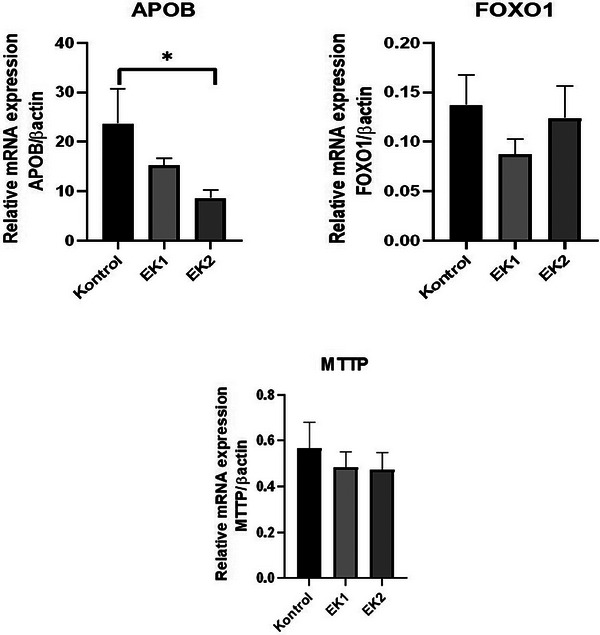
Expression levels of VLDL (very low‐density lipoprotein triglyceride) genes mRNA in the control, EK1 and EK2 groups. *, p<0,05

In our study, involved VLDL mRNA gene expression was investigated in the control, EK1 and EK2 groups. Expression levels of metabolic genes decreased in *APOB, FOXO1* and *MTTP* genes in the EK1 group compared to the control group (Figure 2). *APOB* mRNA gene expression was significantly decreased in the EK2 group compared to the control group (*p* < 0.05) (Figure 2). *FOXO1* mRNA gene expression showed a decreasing change in the EK1 group compared to the control and EK2 groups (*p* > 0.05) (Figure 2). In the EK2 group, all of metabolic genes were downregulated compared to the control group (Figure 2). In the *FOXO1* gene, the EK2 group was upregulated compared to the Ek1 group, unlike all other genes (Figure 2).

### 3.3 Antioxidative Gene Expression

**FIGURE 3:**
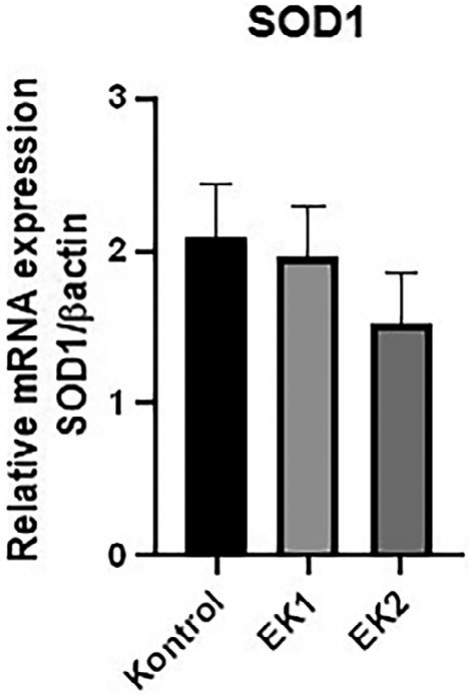
Expression levels of antioxidative gene mRNA in the control, EK1 and EK2 groups.

In our study, involved antioxidative mRNA gene expression was investigated in the control, EK1 and EK2 groups. Expression levels of antioxidative (*SOD1*) genes decreased in the EK1 and EK2 groups compared to the control group (Figure 3). However, these decreases in the antioxidative genes group are not statistically significant (*p* > 0.05) (Figure 3).

## 4 Discussion

We analyzed the expression levels of some metabolic genes in the liver by adding a mixture of essential oils (juniper, rosemary, thyme, mint) rich in polyunsaturated fats at different ratios to the drinking water of geese. Studies comparing gene expression of essential oil intake in different groups have generally been by conducted in fattening studies (Gumus et al. 2017; Tang et al. 2018).

The *CYP2C* overexpression in fatty liver has shown that liver function is injured and that the detoxification process is impaired (Zhu et al. 2011). In this study, essential oil added to drinking water significantly reduced *CYP2C19* expression in the fatty livers of geese, indicating that essential oils were less harmful to liver function in geese. This is consistent with other study in which essential oil was added to feed (Tang et al. 2018). *CYP2K1*, which plays a role in linoleic acid metabolism, was also downregulated in the livers of geese that drank supplemented essential oil compared to those of non‐drinking geese. This suggests that they contribute to the protection of hepatic unsaturated fatty acids against oxidation caused by xenobiotics (Tang et al. 2018). *ELOVL1, ELOVL2* and *ACSBG2* genes, which are involved in fatty acid biosynthesis, play an important role in lipid metabolism (Tang et al. 2018). *ELOVL1* gene is closely associated with improved liver production performance of geese (X. Liu et al. 2020). Compared to control group geese, *ELOVL* was all downregulated and *ACSBG2* remained approximately stable in geese drinking essential oil substituted water. This shows that essential oil supplementation to water can increase intracellular fatty acid synthesis and lipid formation.

*SOD1* is a Cu/Zn enzyme responsible for catalyzing the disproportionation of superoxide into hydrogen peroxide and dioxygen (Sea et al. 2015). *SOD1*‐deficienct mice are prone to developing fatty liver (Lee et al. 2015). Consistent with other work (Tang et al. 2018), in this study, the downregulation of *SOD1* in geese drinking essential oil‐supplemented water may affect peroxisomal fatty acid oxidation and contribute to increased lipid accumulation in the liver.

Lipid secretion and distribution is a central reason to maintain lipid homeostasis. *MTTP* gene is an essential gene for the assembly and incorporation of recently designed lipoprotein particles into the system (D. D. Liu, Han, et al. 2016). In our study, *MTTP* gene regulation was downregulated, although not significantly. It was found that interfering with *FoxO1* gene can inhibit the differentiation of fat cells, and in another study, silencing *FoxO1* inhibited the differentiation of fat cells and the growth of lipid droplets (L. Liu, Zheng, et al. 2016; Munekata and Sakamoto 2009). Our study was seen that the expression of *FoxO1*, a transcription factor that plays a key role in hepatic insulin signalling, was downregulated by 0.4 mL and upregulated 0.8 mL essential oil mixture. Foxo1 gene expression was upregulated as the essential oil ratio increased, but our results were not statistically significant. Similar situations have been detected in studies with essential oil in geese (Pan et al. 2011a, 2011b).

*APOB* is a protein found on the surface of cholesterol particles. It contains fats that are particularly harmful for vascular health. If the *APOB* level is high, even if the cholesterol level is normal, it poses a risk for cardiovascular diseases (Behbodikhah et al. 2021). According to the results obtained in our study, increasing the intake of essential oils with food decreased APOB gene expression in the liver (Figure 2), and it is thought that essential oils intake in cholesterol‐related diseases in humans will reduce cholesterol and reduce cardiovascular diseases.

## 5 Conclusion

In our study, essential oil supplementation to drinking water reduced *APOB* gene expression in the liver, and it is thought that essential oil intake will reduce cardiovascular diseases by lowering cholesterol in cholesterol‐related diseases. Differential expression of lipid metabolism‐related genes was determined between groups treated with essential oils (juniper, rosemary, thyme, mint).

## Author Contributions

Özlem Durna and Mustafa Hitit contributed to the study conception and design. Material preparation, data collection and analysis were performed by Özlem Durna, Mustafa Hitit, Zafer Usta and Gültekin Yıldız. The first draft of the manuscript was written by Zafer Usta, and all authors commented on the manuscript. All authors read and approved the final manuscript.

## Ethics Statement

Investigation of the effect of the addition of essential oils obtained from different aromatic plants at certain ratios to the drinking water of the Kars province domestic breed geese on some metabolic genes with the code KAU‐HADYEK/2019‐128, which was evaluated and approved by the Caucasus University Animal Experiments Local Ethics Committee (KAÜ‐HAYDEK). The research titled “investigation of the effects on gene pathways” was planned in accordance with the principles of the KAU‐HADYEK directive, and that the project is ‘APPROPRIATE’ in terms of animal use and legislation, to be carried out on 18 poultry (goose liver samples) (animals in the study with approval number 2018–054) with a duration of 6 months. It was decided unanimously.

## Conflicts of Interest

The other authors declare no conflicts of interest.

### Peer Review

The peer review history for this article is available at https://www.webofscience.com/api/gateway/wos/peer‐review/10.1002/vms3.70285.

## Data Availability

The datasets generated during and/or analyzed during the current study are available from the corresponding author on reasonable request.
